# Auxin Treatment Enhances Anthocyanin Production in the Non-Climacteric Sweet Cherry (*Prunus avium* L.)

**DOI:** 10.3390/ijms221910760

**Published:** 2021-10-05

**Authors:** Daniel Clayton-Cuch, Long Yu, Neil Shirley, David Bradley, Vincent Bulone, Christine Böttcher

**Affiliations:** 1 Adelaide Glycomics, Waite Campus, School of Agriculture, Food and Wine, University of Adelaide, Adelaide, SA 5064, Australia; daniel.clayton-cuch@adelaide.edu.au (D.C.-C.); long.yu@adelaide.edu.au (L.Y.); proteus01@bigpond.com (N.S.); 2 Commonwealth Scientific and Industrial Research Organisation (CSIRO), Waite Campus, Glen Osmond, SA 5064, Australia; 3 Agilent Technologies Australia Pty Ltd., Mulgrave, Melbourne, VIC 3170, Australia; david.bradley@agilent.com; 4 Department of Chemistry, Division of Glycoscience, Royal Institute of Technology (KTH), School of Engineering Sciences in Chemistry, Biotechnology and Health, AlbaNova University Centre, 10691 Stockholm, Sweden

**Keywords:** anthocyanin, auxin, ethylene, ripening, sweet cherry (*Prunus avium*)

## Abstract

Abscisic acid (ABA) is a key signaling molecule promoting ripening of non-climacteric fruits such as sweet cherry (*Prunus avium* L.). To shed light on the role of other hormones on fruit development, ripening and anthocyanin production, the synthetic auxin 1-naphthaleneacetic acid (NAA) was applied to sweet cherry trees during the straw-color stage of fruit development. NAA-treated fruits exhibited higher concentrations of 1-aminocyclopropane-1-carboxylic acid (ACC) and ABA-glucose ester (ABA-GE), which are a precursor of ethylene and a primary storage form of ABA, respectively. Consistent with these observations, transcript levels of genes encoding ACC synthase and ACC oxidase, both involved in ethylene biosynthesis, were increased after 6 days of NAA treatment, and both ABA concentration and expression of the regulator gene of ABA biosynthesis (*NCED1* encoding 9-cis-epoxycarotenoid dioxygenase) were highest during early fruit ripening. In addition, transcript levels of key anthocyanin regulatory, biosynthetic and transport genes were significantly upregulated upon fruit exposure to NAA. This was accompanied by an increased anthocyanin concentration and fruit weight whilst fruit firmness and cracking index decreased. Altogether our data suggest that NAA treatment alters ethylene production, which in turn induces ripening in sweet cherry and enhanced anthocyanin production, possibly through ABA metabolism. The results from our study highlight the potential to use a single NAA treatment for manipulation of cherry ripening.

## 1. Introduction

Fruit species can be classified into two main groups depending on their pattern of ripening. In climacteric fruits, ripening is initiated by the gaseous hormone ethylene and continues post-harvest, whereas non-climacteric fruits ripen independently of ethylene [1]. Sweet cherry (*Prunus avium* L.) is an economically important horticultural crop cultivated in temperate climates across the globe and reported to be a non-climacteric fruit. Cherry ripening is accompanied by changes in color, sugar, organic acid and vitamin content, which ultimately determine the quality of the fruit [2]. Of all quality attributes, size, color and sweetness are most critical as they drive consumer acceptance and, ultimately, the price of cherry fruits [3,4]. In addition to its visual impact, cherry color relates to the nutritional value of the fruit, as it is derived from the accumulation of health-promoting anthocyanins, a class of flavonoids [5,6]. These water-soluble pigments are responsible for the blue, purple and red colors of flowers, fruits and vegetables. Many genes involved in the complex biosynthetic pathways or transcriptional regulation of flavonoid production have been isolated and characterized in model plants such as maize, Arabidopsis, petunia and snapdragon [7], as well as in several horticultural crops including apple, grape and mangosteen [5,8,9,10]. However, less is known about the hormonal regulation of anthocyanin biosynthesis. Past research has established abscisic acid (ABA) as a central signaling molecule for non-climacteric fruit ripening. Indeed, this hormone regulates fruit softening, color change through anthocyanin accumulation, and other compositional modifications during ripening [11,12,13]. In addition to ABA, other phytohormones such as auxins, jasmonic acid (JA), gibberellins (GAs) and cytokinins are involved in the ripening of non-climacteric fruit. Regulatory effects of these hormones include increasing fruit size through initiation of cell division and expansion, and the exogenous application of synthetic auxins in particular has resulted in increased fruit weight and size in a variety of fruit species [14,15,16]. However, the precise role of auxins in the regulation of fruit ripening remains unclear as both ripening delay and advancement caused by the application of auxins have been reported in climacteric and non-climacteric fruit species [17]. Despite these conflicting observations, the promotion of ripening by auxin analogues occurs most frequently in climacteric fruit and has been associated with increased ethylene formation resulting from auxin-induced stimulation of the ethylene biosynthesis pathway [17]. In grapevine, which is a non-climacteric fruit species with similar ripening characteristics to sweet cherry, a delay in sugar and anthocyanin accumulation and an increase in fruit size have been observed in different cultivars following the application of natural or synthetic auxins [18,19,20].

To better understand the role of auxins in the maturation of sweet cherry, we have analyzed how 1-naphthaleneacetic acid (NAA), a synthetic auxin used as a Plant Growth Regulator in many horticultural crops [21], alters cherry ripening and affects anthocyanin concentration, fruit weight and firmness, as well as the cracking index. Further investigation of the molecular mechanisms by which auxin treatment might induce anthocyanin accumulation in cherry fruit revealed interactions between auxin, ethylene and ABA pathways as possible contributors to this response.

## 2. Results

### 2.1. Fruit Maturity Parameters in Response to the Application of NAA

To test the effect of auxin on the ripening of non-climacteric sweet cherry fruit, trees at the straw-color stage of fruit ripening were sprayed with a single application of the synthetic auxin NAA. NAA-treated cherries collected 2 days prior to the commercial harvest of untreated fruit in the same block had unchanged total soluble solids (TSS), total acids, and pH compared to control fruit (Table 1). An increase in fruit weight and a decrease in firmness was observed in treated fruit, with the latter being indicative of a ripening advancement. Treatment with NAA affected the fruit skin color coordinates (Table 1) as significantly lower L*, a*, b*, hue angle and chroma values were measured for NAA-treated fruit, which are associated with increased anthocyanin concentrations [22,23].

### 2.2. Increased Anthocyanin Accumulation in Response to the Application of NAA

**Figure 1:**
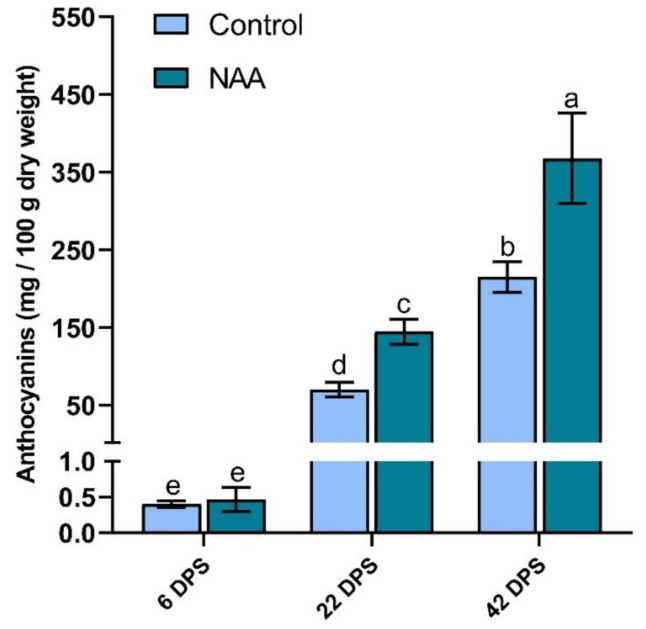
LC-MS/MS analysis of total anthocyanin content in sweet cherry (*Prunus avium* L.) fruit at three timepoints after the pre-ripening application of 100 mg/L NAA. Data represent means ± standard error (*n* = 3); bars are denoted by a different letter (a, b, c, d, e) if the means differ significantly (*p* < 0.0001) using one-way ANOVA followed by Waller-Duncan’s post hoc test. DPS = days post spray.

After determination of the anthocyanin profile of sweet cherry fruit (Figure S1), anthocyanin accumulation after NAA treatment was analyzed by LC-MS/MS 6, 22 and 42 days post-spray (dps) and compared to anthocyanin content in untreated control fruits at the same timepoints. Anthocyanin concentration increased with time, and at the last two timepoints was significantly higher in NAA-treated cherries than in untreated fruits (Figure 1). The highest concentration of anthocyanins was in NAA-treated fruit at 42 dps (368 mg/100 g dry weight), which was 71% higher than in untreated fruit at the same timepoint (215 mg/100 g dry weight; Figure 1).

### 2.3. Differential Expression of Anthocyanin Regulatory and Biosynthetic Genes in Response to NAA Treatment

**Figure 2:**
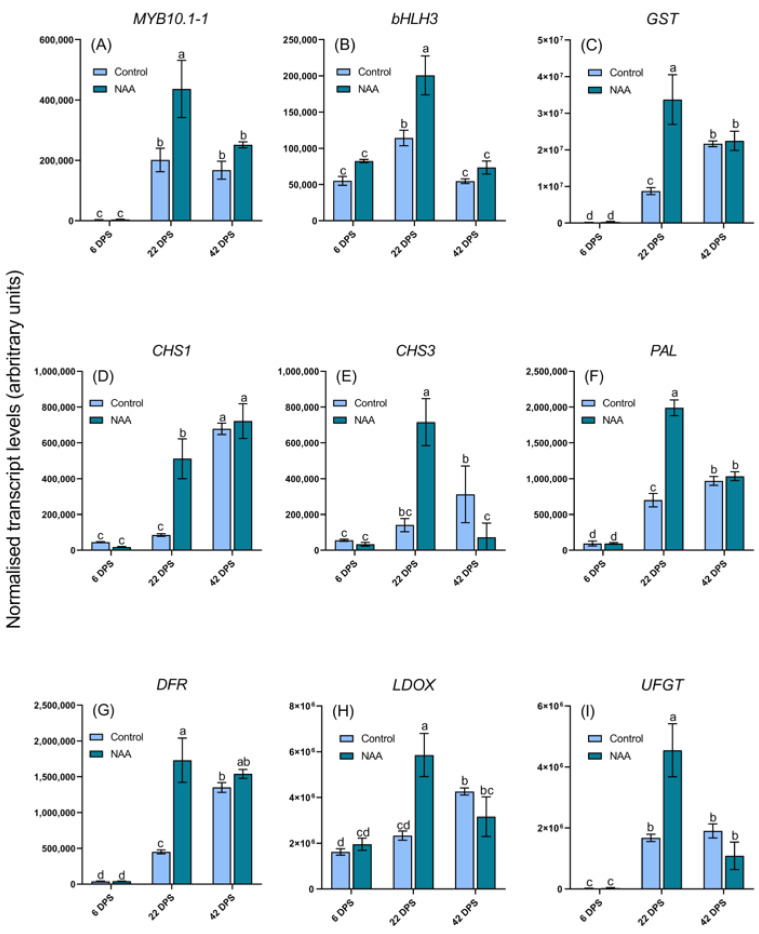
qRT-PCR analysis of anthocyanin regulatory genes in sweet cherry (*Prunus avium* L.) fruit at three timepoints after the pre-ripening application of 100 mg/L NAA. (**A**) *MYB10.1-1 p* < 0.0001, (**B**) *bHLH3 p* < 0.0001 and anthocyanin structural genes (**C**) *GST p* < 0.0001, (**D**) *CHS1 p* <0.0001, (**E**) *CHS3 p* < 0.0001, (**F**) *PAL p* < 0.0001, (**G**) *DFR p* < 0.0001, (**H**) *LDOX p* < 0.0001 and (**I**) *UFGT p* < 0.0001. All data represent geometric means ± s. e. (*n* = 3), bars are denoted by a different letter (a, b, c, d, bc, cd) if the means differ significantly using one-way ANOVA followed by Waller-Duncan’s post hoc test. DPS = days post spray.

To further investigate the potential inductive effect of NAA on the production and/or accumulation of anthocyanins, the expression of anthocyanin regulatory and biosynthetic genes was assayed (Figure 2). MYB-bHLH-WD repeat (MBW) complexes regulate the transcription of anthocyanin biosynthesis genes [26], and both bHLH and MYB transcription factors are encoded by large gene families. Previous studies have identified MYB10.1-1 and bHLH3 as playing critical roles in the fruit coloration of sweet cherry [27], so the expression levels of the corresponding genes were analyzed in this study (Figure 2). *MYB10.1-1* expression was significantly upregulated following NAA application at both the 22 and 42 dps timepoints, with 118% and 48% increases, respectively, compared with expression in untreated fruit at the same timepoints (Figure 2A). *bHLH3* had significantly increased expression in NAA-treated cherries at 22 dps, with 76% increased expression level (Figure 2B).

The anthocyanin-related glutathione S-transferase gene (*GST*) has been shown as essential for anthocyanin transport into the vacuole and therefore coloration in many climacteric and non-climacteric fruits [28]. *GST* expression was significantly upregulated at 22 dps in NAA-treated fruit, with a 291% increased expression level (Figure 2C). The expression of six anthocyanin structural biosynthetic genes (*PAL, CHS1, CHS3, DFR, LDOX* and *UFGT*) was at low levels during early fruit development (Figure 2), however expression levels increased from 22 dps, in accordance with the pattern of anthocyanin accumulation during ripening (Figure 1 and Figure 2), which is consistent with previous gene expression studies in sweet cherry [29,30,31,32]. All six anthocyanin biosynthesis genes were significantly upregulated in the NAA-treated fruit at the 22 dps timepoint, indicating significant upregulation of the entire anthocyanin biosynthesis pathway in response to NAA treatment, particularly during the middle stage of fruit development when anthocyanin biosynthesis begins (Figure 2).

### 2.4. Altered Hormone Levels of Cherry Fruit in Response to the Application of NAA

**Figure 3:**
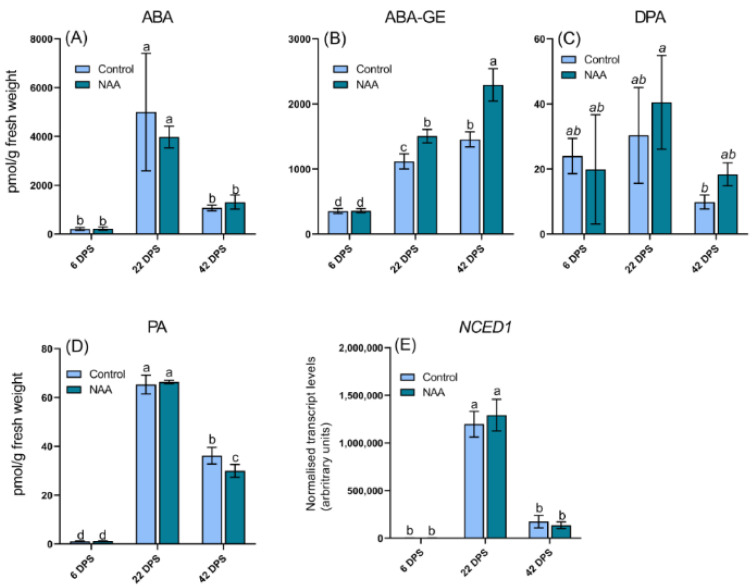
LC-MS/MS analysis of abscisic acid (ABA) and ABA-related compounds concentrations in sweet cherry (*Prunus avium* L.) fruit at three timepoints after the pre-ripening application of 100 mg/L NAA are shown including (**A**) ABA *p* = 0.0002, (**B**) abscisic acid-glucose ester (ABA-GE) *p* < 0.0001, (**C**) dihydrophaseic acid (DPA) *p* = 0.075 ns and (**D**) phaseic acid (PA) *p* < 0.0001. qRT-PCR data are also shown for the ABA biosynthesis gene (**E**) *NCED1 p* < 0.0001. All data represent means ± s. e. (*n* = 3), bars are denoted by a different letter (a, b, c, ab) if the means differ significantly using one-way ANOVA followed by Waller-Duncan’s post hoc test. DPS = days post spray.

**Figure 4:**
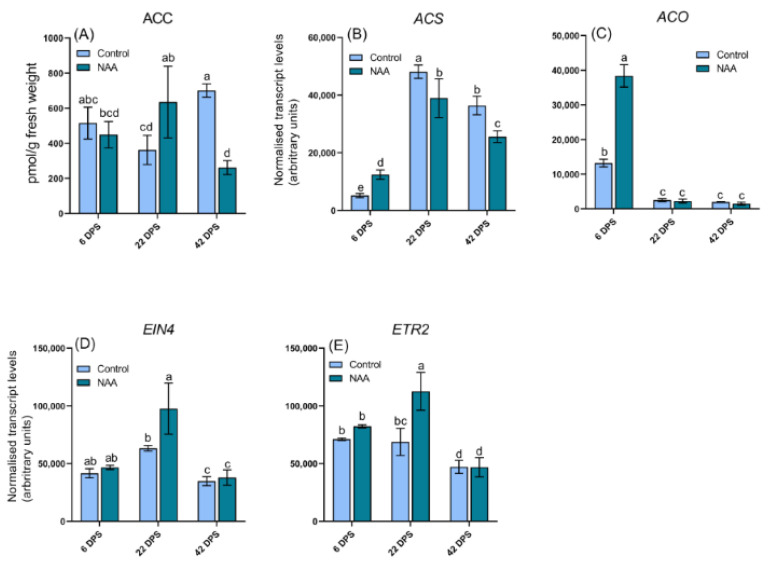
LC-MS/MS analysis of 1-aminocyclyopropane-1-carboxylic acid (ACC) concentrations in sweet cherry (*Prunus avium* L.) fruit at three timepoints after the pre-ripening application of 100 mg/L NAA (**A**) ACC *p* = 0.0021. Additionally, shown is qRT-PCR data of ethylene biosynthetic and receptor genes (**B**) *ACS p* <0.0001, (**C**) *ACO p* <0.0001, (**D**) *EIN4 p* < 0.0001 and (**E**) *ETR2 p* < 0.0001. All data represent means ± s. e. (*n* = 3), bars are denoted by a different letter (a, b, c, d, e, ab, bc, cd, abc, bcd) if the means differ significantly using one-way ANOVA followed by Waller-Duncan’s post hoc test. DPS = days post spray.

**Figure 5:**
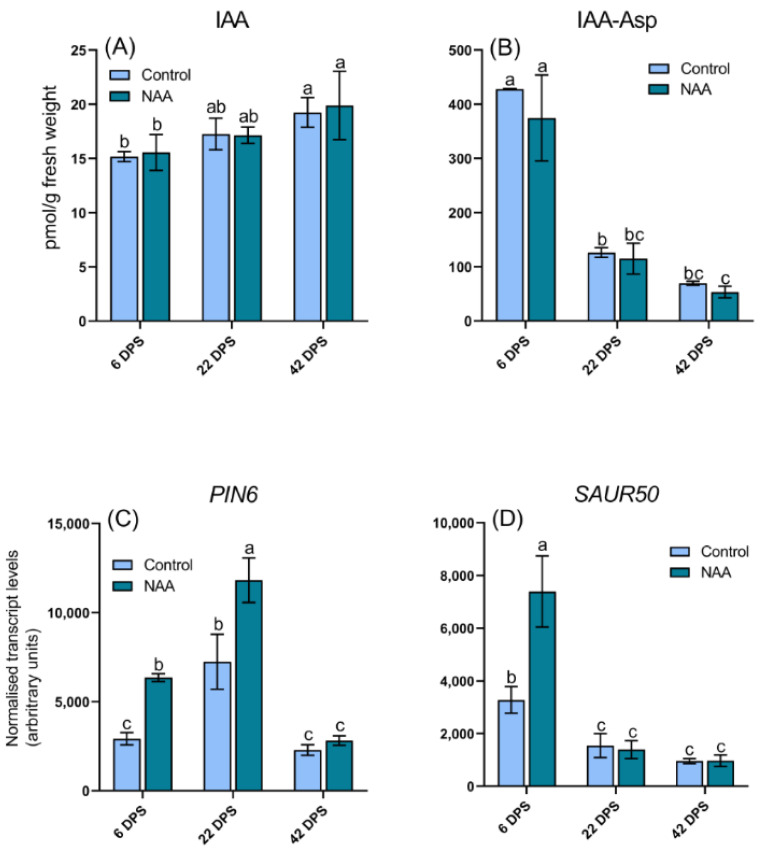
LC-MS/MS analysis of endogenous auxin concentrations in sweet cherry (*Prunus avium* L.) fruit at three timepoints after the pre-ripening application of 100 mg/L NAA are shown including (**A**) indole-3-acetic acid (IAA) *p* = 0.0290 and (**B**) indole-3-acetic acid aspartate (IAA-Asp) *p* < 0.0001. qRT-PCR analysis is also shown for of auxin regulatory genes (**C**) *PIN6 p* < 0.0001 and (**D**) *SAUR50 p* < 0.0001. All data represent geometric means ± s. e. (*n* = 3), bars are denoted by a different letter (a, b, c, ab, bc) if the means differ significantly using one-way ANOVA followed by Waller-Duncan’s post hoc test. DPS = days post spray.

A range of hormones have been reported to regulate ripening in non-climacteric fruits including ABA, auxin, JA, GA and to a lesser extent ethylene [17]. To investigate if the increased production of anthocyanins in response to NAA treatment was mediated by auxin-induced changes to other hormone pathways, such as ABA and ethylene, the concentrations of these hormones and their main conjugates were quantified by LC-MS/MS (Figure 3, Figure 4 and Figure 5). No differences in ABA concentrations were observed between the control and NAA-treated cherry fruit (Figure 3A), however a significantly increased concentration of ABA-GE, the primary inactive storage form of ABA in plants [33], was detected at the 22 and 42 dps timepoints in NAA-treated fruits (Figure 3B).

The two main catabolites of ABA, phaseic acid (PA) and dihydrophaseic acid (DPA), were found at similar levels in control and NAA-treated fruits, with the only significant difference detected at 42 dps, where the PA concentration in control cherries was slightly higher than in NAA-treated cherries (Figure 3C,D).

Ethylene concentration is difficult to measure directly as it is a gas, so the concentration of its precursor ACC was measured as a proxy to assess changes in ethylene levels, as reported elsewhere [34]. A significant increase in ACC concentration was measured at the 22 dps timepoint in NAA-treated fruits, however this was followed by a significant decrease in ACC at the 42 dps timepoint compared with control fruits (Figure 4A). No significant differences in the concentrations of either the primary plant auxin hormone IAA (Figure 5A) or the auxin degradation product, IAA-Asp (Figure 5B), were observed at any timepoints. NAA concentration in the pericarp decreased over the course of the ripening phase (Figure S2), but was still detected at 124 ± 21 pmol/g fresh weight at the point of harvest (42 dps). Conversely no NAA was detected in the control samples. While jasmonic acid (JA) has been suggested to promote anthocyanin biosynthesis in cherry [35], the concentrations of JA and its active conjugate jasomonic acid-isoleucine (JA-Ile) in the present study were below the quantification limit (data not shown).

### 2.5. Differential Expression of Hormone Related Genes in Response to NAA Treatment

The expression of hormone signaling and biosynthesis genes was also analyzed to understand how the differences in hormone levels may be regulated (Figure 3, Figure 4 and Figure 5). To examine the response to exogenous NAA, we first investigated key auxin-responsive genes. Significantly higher expression levels of the auxin transport gene *PIN-FORMED 6* (*PIN6*) were observed in NAA-treated fruit, especially at the early timepoints (Figure 5C). *PIN6* is an efflux carrier facilitating the direction of auxin flow, therefore playing a vital role in the transport of auxin within plant tissues [36]. *SMALL AUXIN UP RNAs* (*SAURs*) are the largest family of early auxin response genes [37]; *SAUR50* showed significantly increased expression at early timepoints in NAA-treated fruit, with a 127% increase at 6 dps (Figure 5D).

Genes involved in ethylene biosynthesis and signaling were investigated and found to be differentially expressed in response to NAA (Figure 4B–E). The ethylene biosynthesis pathway consists of two dedicated steps, the first step is facilitated by aminocyclopropane-1-carboxylate synthase (*ACS*) and the second is mediated by aminocyclopropane-1-carboxylate oxidase (*ACO*) [38]. The biosynthetic genes *ACS* and *ACO* were both upregulated in response to NAA at 6 dps, but at later timepoints, *ACS* was down-regulated by comparison to levels in untreated fruit, while *ACO* expression decreased to extremely low levels in both treated and control fruit (Figure 4B,C). Genes encoding two putative ethylene receptors, *EIN4* and *ETR2*, were also found to have altered expression in response to NAA treatment, with significantly upregulated expression at 22 dps in NAA-treated fruit (Figure 4D,E).

In accordance with the similar ABA concentrations measured in control and NAA-treated fruit, there were no significant differences in expression levels of *NCED1*, the gene encoding 9-cis-epoxycarotenoid dioxygenase, which regulates the rate limiting step in ABA biosynthesis, between NAA-treated or control fruit (Figure 3E) [33]. Although there were no significant differences in ABA concentrations or the expression level of *NCED1* between NAA-treated and control fruit, there were differences across development. Both ABA concentrations and *NCED1* expression levels were highest at the 22 dps timepoint, which is during early fruit ripening. A peak in ABA concentrations and therefore *NCED1* transcript levels have been previously reported in sweet cherry at the start of fruit ripening [12,13,32].

## 3. Discussion

Plant hormones are important regulatory signals of fruit ripening controlling many aspects of development in both climacteric and non-climacteric fruit [39]. For this reason, they have been used extensively to manipulate fruit development in various plant species [17]. The synthetic hormone NAA is the compound of choice in applications involving auxins due to its low toxicity and superior metabolic stability compared with naturally occurring auxins or their precursors, such as indole 3-butyric acid (IBA) [40,41,42]. Like IBA, NAA and combinations of IBA and NAA are commonly used as rooting agents and have been shown to exert positive effects on fruit weight and composition in various horticultural crops [43]. In our study the application of NAA to sweet cherry trees during the straw-color stage of fruit ripening resulted in increased fruit weight and anthocyanin concentration, whilst reducing cracking index and fruit firmness (Table 1, Figure 1). The increased weight observed in NAA-treated cherry fruit was expected as the same response has been previously reported in studies of both climacteric and non-climacteric fruit species in response to exogenous auxin application [44,45]. However, decreased fruit firmness and significantly increased total anthocyanin concentrations were unexpected as they are indicative of accelerated ripening, contradicting the ripening-delaying effect of auxin treatments commonly observed in non-climacteric fruit [18,19,46]. One study observed a similar response in sweet cherry treated with the synthetic auxin 2,4-DP in combination with NAA at lower concentrations than in the research reported here [45]. Sweet cherry fruit treated with NAA showed signs of enhanced ripening through improved color, allowing harvest four days earlier compared to the control fruit [45]. In our experiments, the concentration of NAA measured from 6–42 dps (458–124 pmol/g fresh weight, Figure S2) was well above endogenous auxin levels; hence, it can be assumed that NAA was present in the fruit at physiologically active concentrations through the ripening phase. The high rate of applied NAA (100 mg/L) implies that only a small proportion of NAA was taken up by the fruit, that it was transported out of the fruit tissue, or that conversion into other metabolites occurred within the fruit. A high concentration of NAA-Asp in NAA treated cherries at harvest (4236 pmol/g fresh weight) indicated that formation of this main NAA metabolite [47] was a major contributor to the decrease and maintenance of the relatively low observed NAA concentration in cherries as has previously been suggested for NAA-treated grapes [48]. As auxins have been suggested to inhibit anthocyanin production in diverse crops [18,19,20,49,50,51] our data indicate that the observed increased anthocyanin concentration upon NAA treatment of sweet cherry is mediated by compounds not related to auxins.

The NAA-treatment of pre-ripening cherries stimulated ethylene biosynthesis as inferred by a significant increase in the concentration of the ethylene biosynthesis precursor ACC and increased expression of ethylene biosynthesis genes (*ACS, ACO*) at the first sampling timepoint after the auxin treatment (6 dps). This was followed by a decrease towards the end of ripening (42 dps) (Figure 4), indicative of an auxin-induced activation of ethylene biosynthesis and subsequent homeostatic response restoring normal ethylene levels. This was further evidenced by an upregulation of the expression of *EIN4* and *ETR2* at 22 dps in NAA-treated fruit, consistent with suppressed ethylene signaling in response to an increase in ethylene concentration in the fruit. Interactions between auxins and ethylene were first described by Morgan and Hall, reporting 2,4-DP stimulated ethylene biosynthesis in cotton plants [15]. It has since been demonstrated that the application of synthetic auxins can directly induce *ACS* and *ACO* expression in many climacteric and non-climacteric fruit species such as apple, pear, peach and grape [14,16,52]. The findings reported here support the notion that auxin is capable of inducing an ethylene biosynthesis response in sweet cherry and suggest ethylene as a possible inducer of ripening in sweet cherry.

Yet, the role of ethylene in sweet cherry ripening remains ambiguous. Hartmann (1989) reported that ethylene could be the trigger for sweet cherry maturation, since its levels increased around the time of maturation. In addition, the application of 25–250 mg/L of the ethylene releasing compound ethephon 28 days after full bloom has been reported to promote sweet cherry fruit ripening [53]. Other reports show ABA and ethylene working synergistically to induce the ripening of sweet cherry and therefore anthocyanin accumulation [12]. Conversely, there are studies recording no increase in ethylene during sweet cherry ripening. Ethephon treatment (10–1000 mg/L) did not stimulate respiration during cherry fruit development in one report, in line with sweet cherry being non-climacteric [54]. Accordingly, it remains to be determined whether ethylene is a main regulator of the maturation of sweet cherry.

However, for one ripening parameter, the biosynthesis of anthocyanins, there is convincing evidence for the inductive effects of ethylene from other non-climacteric species such as grapes. A study showed that the transcript levels of the anthocyanin biosynthetic genes *CHS*, *F3H* and *UFGT* were enhanced by 30% within 6 h following gassing with ethylene in wine grapes [55]. In addition, researchers described an ethylene-responsive element within the grapevine *UFGT* gene promoter, although no functional data were provided on its activity [56]. As shown in Figure 2, transcript levels of key anthocyanin regulatory, biosynthesis and transport genes were significantly upregulated at 22 dps in NAA-treated fruit, which is consistent with the significantly increased concentration of ACC at the same timepoint and an indication that ethylene may induce anthocyanin biosynthesis in sweet cherry during fruit ripening.

Previous reports have provided evidence that increased biosynthesis of ethylene can trigger ABA biosynthesis in some non-climacteric species [57,58,59] and ABA has been established as an inducer of anthocyanin biosynthesis in many plant species such as sweet cherry [35,59]. In addition, MYB has been shown to be regulated by ABA levels and transient promoter assays have demonstrated that MYB interacts with several anthocyanin-related bHLH transcription factors to activate the promoters of structural genes involved in anthocyanin biosynthesis [35]. It is therefore plausible that the auxin-induced increase in ethylene concentrations may have enhanced the biosynthesis of ABA which further promoted anthocyanin biosynthesis. A similar stimulation of ABA biosynthesis by auxin via the ethylene pathway was first observed upon treatment with auxin herbicides and it has since been suggested that the auxin-ethylene-ABA cascade plays a role in different stress responses of plants [60]. Although the results from our study showed no direct evidence of increased ABA concentrations or biosynthesis, the significantly higher concentrations of ABA-GE in NAA-treated fruit at both the 22 and 42 dps timepoints (Figure 3B) indicated an auxin-induced stimulation of ABA biosynthesis between 6 and 22 days after the NAA treatment. ABA-GE has been suggested to serve as a storage/transport form of ABA, or considered a final inactive product of ABA catabolism [61]. More recent evidence has suggested that ABA-GE can be hydrolyzed to form the active free ABA compound, suggesting pathways exist to rapidly increase ABA levels from inactive ABA-GE [62]. Higher concentrations of ABA-GE in NAA-treated fruit may indicate that auxin application, in addition to directly stimulating the ethylene biosynthesis pathway, also led to activation of the ABA biosynthesis pathway, either directly, or mediated by ethylene, which ultimately promoted anthocyanin biosynthesis.

In summary, the application of NAA to sweet cherry during the straw-colored stage of fruit development altered fruit parameters such as size, colour and cracking susceptibility and modified the fruit hormone profile. Our data provide evidence that ethylene could possibly act as an inducer of ripening in sweet cherry, even though sweet cherry is considered a non-climacteric species. Whether ethylene plays a direct role in modulating sweet cherry ripening or mediates this through inducing ABA biosynthesis remains unclear and will require further research. The potential for a single NAA treatment of sweet cherry fruit at the straw-colored stage of development to increase anthocyanin concentrations and decrease cracking susceptibility should open consideration for its use in cherry ripening manipulation.

## 4. Materials and Methods

### 4.1. Fruit Material and Auxin Application

The auxin application experiment was conducted in 2019 on ‘Sweet Georgia’ (*Prunus avium* L.) trees grafted on Colt rootstocks in a commercial orchard at Lenswood, South Australia.

Gro-Chem NAA 20 (Sumitomo Chemical Australia, Epping, Australia) was diluted in water to 100 mg/L NAA and applied in 0.01% Viti-wet (SST Australia, Dandenong South, Australia) at a rate of 1875 L/ha with a commercial sprayer at straw-colour stage (27 November 2019) to 10 trees in a north/south-facing row. The NAA concentration of 100 mg/L was chosen as it lies within the concentration range that has successfully been used to delay the ripening of wine grapes [16,17,18]. Leaving a gap of three trees, 10 control trees were sprayed in the same way with a 0.01% Viti-wet solution. Three replicates of 25 cherries each were randomly sampled from the NAA-treated and control trees at 6, 22 and 42 days post spray (dps), immediately frozen in liquid nitrogen and stored at −80 °C. The three sampling timepoints correspond to stage II/III transition, early stage III and late stage III cherry growth stages (Turkey and Young, 1939). After removal of pedicels and pits, the frozen pericarp tissue was ground to a powder using an IKA A11 basic analytical mill (IKA, Staufen, Germany), stored at −80 °C and used for anthocyanin, hormone and RNA extractions. Two days prior to the commercial harvest of the fruit on 13 January 2020 (42 dps), three replicates of approximately 100 unblemished cherries were collected from both control and NAA-treated cherry trees, transported to the laboratory on ice and subjected to fruit quality measurements.

### 4.2. Fruit Maturity Parameters at Harvest

A subsample of 30 cherries/replicate was used to determine the average fruit weight using a precision digital balance (PB3002-S; Mettler-Toledo, Melbourne, Australia). Juice from the same fruit was obtained by squeezing through muslin cloth and analyzed for total soluble solids (TSS) (RFM710 digital refractometer; Bellingham Stanley, Tunbridge Wells, United Kingdom). Acid content and pH from 5 mL aliquots of the juice were measured with an auto-titrator (855 Robotic Titrosampler; Metrohm, Gladesville, Australia). Titration was performed with 0.1 N NaOH (Rowe Scientific, Wangara, Australia) to pH 8.1, and total acid was expressed as malic acid equivalents. Firmness and fruit diameter of a second subsample of 25 cherries/replicate were determined with a FirmTech instrument (FirmTech 500; BioWorks, Wamego, KS, USA). The same subset of cherries was used to monitor fruit colour by obtaining CIE 1976 (L*, a*, b*) coordinates from two sides of the mid-section of each cherry with a chromameter (CR-200; Minolta, Osaka, Japan) and averaging the two measurements. Chroma and hue angle were calculated from the a* and b* colour coordinates [25]. A third subsample of 30 cherries/replicate was subjected to a cracking test by immersion in distilled water for 6 h at 20 °C. Cracked fruit were counted every 2 h and removed from the immersion liquid. The cracking index was calculated according to [63].

### 4.3. Analysis of Anthocyanins by LC-MS/MS

Lyophilized pericarp tissue (250 mg) of cherry was extracted with 5 mL of 70% aqueous methanol (*v*/*v*) containing 0.5% trifluoroacetic acid. Tubes were wrapped in aluminum foil to keep dark and the mixture was placed on a PTR-35 Multi-Rotator (Grant Instruments, Shepreth, United Kingdom) for 2 h at ambient temperature. After centrifugation at 4 °C at 10,000× *g* for 10 min (Eppendorf 5810 R, Darmstadt, Germany), supernatant was removed and stored at −20 °C.

LC-MS/MS analysis of the anthocyanin extract was performed on an Agilent 1290 Infinity II HPLC (Agilent, Santa Clara, CA, USA) coupled with an Agilent 6495 Triple Quad mass spectrometer equipped with an electrospray ionization source (Agilent Jet Stream ion source). Instrument control and data acquisition were performed using MassHunter workstation software (LC/MS Data Acquisition, version B.09.00, Agilent) while processing was done using MassHunter quantitation software (Quantitative Analysis, version B.09.00, Agilent). An Agilent InfinityLab Poroshell 120 SB-C18 (2.1 × 100 mm, 2.7 µm) column was used at a controlled temperature of 30 °C. Samples were eluted with a gradient of 5% formic acid in water (eluent A) and acetonitrile (eluent B) at a flow rate of 0.3 mL/min with an injection volume of 5 µL. Elution conditions were 5% B (10 s), a gradient from 5% to 90% B (10 s–19 min), a gradient from 90% to B (19–21 min), and finally 5% B (21–24 min). Mass spectra were recorded in multiple reaction monitoring (MRM) mode. LC-MS/MS source parameters and fragment ions are shown in Table S1**.**

### 4.4. Analysis of Hormones by LC-MS/MS

The extraction and analysis of ACC, JA, JA-Ile, IAA, IAA-Asp, AB and its main metabolites, ABA-GE, PA and DPA was based on methods described by [64]. Fifty mg of pericarp tissue was extracted in 1 mL of ice-cold 50% (*v*/*v*) aqueous acetonitrile spiked with deuterated standards: 100 pmol ACC-*d4* (OlChemIm Ltd., Olomouc, Czech Republic), 250 pmol of JA-*d5* (OlChemIm Ltd., Olomouc, Czech Republic), 500 pmol of IAA-*d5* (Cambridge Isotape Laboratories, Andover, USA), IAA-Asp-*d5* synthesized as described by [65] and 5 ng of ABA-*d6*, ABA-GE-*d5*, PA-*d3* and DPA-*d3* (National Research Council Canada, Saskatoon, SK, Canada). The samples were vortexed, sonicated in an ice bath for 5 min and extracted at 4 °C and 2500 rpm for 30 min (Eppendorf MixMate, Macquarie Park, Australia). Samples were centrifuged at 14,000× *g* at 4 °C for 12 min and supernatant transferred to fresh tubes. An HLB-SPE cartridge (30 mg, Waters, Wexford, Ireland), pre-conditioned with 1 mL methanol, 1 mL Nanopore water and 1 mL 50% (*v*/*v*) acetonitrile, was loaded with the 1 mL of sample supernatant. Flow through was collected in glass tubes, and the cartridge was further rinsed with 1 mL 30% (*v*/*v*) acetonitrile. The eluate was combined with the flow through and dried in a vacuum concentrator (SP Genevac miVac, Ipswich, United Kingdom) at 50 °C. The dried sample residue was then resuspended in 50 µL of 30% (*v/v*) acetonitrile; 10 µL of this was used for LC-MS/MS analysis. The extraction and analysis of NAA were performed as described by Böttcher [47].

LC-MS/MS analysis of the plant hormones was performed on an Agilent 1260 Infinity II HPLC (Agilent, Santa Clara, CA, USA) with an Agilent 6470 Triple Quad mass spectrometer equipped with a jet stream ionization source. Instrument control and data acquisition were performed as described above, the column used was a Luna C18 column (75 × 4.6 mm, 5 µm; Phenomenex, Torrance, CA, USA) heated to 50 °C. ABA-related compounds and JA were eluted with a gradient of 0.01% formic acid in water (eluent A) and 0.01% formic acid in 90% (v/c) acetonitrile (eluent B) at a flow rate of 0.5 mL/min and injection volume of 10 μL. Elution conditions were 5% B (5 min), a gradient from 5% to 80% B (5–25 min), 100% B (25–28 min), and 5% B (28–33 min). ACC, IAA and IAA-Asp were eluted with the same A and B eluents at a flow rate of 0.35 mL/min and injection volume of 10 μL. Elution conditions were 0.5% B (3 min), a gradient from 0.5% to 70% B (3–9 min), 100% B (9–13 min), and 0.5% B (13–18 min).

Mass spectra were recorded in MRM mode. To enhance sensitivity of the target analytes, each of the two runs were split into different time segments based on retention times. Time segments were as follows: ACC (1.5 min–3.5 min), IAA/IAA-Asp (3.5 min–10 min), ABA/ABA-GE/PA/DPA (5 min–18.2 min) and JA (18.2 min–21 min)/JA-Ile (21 min–23.5 min). LC-MS/MS run parameters and fragment ions are given in Table S2.

### 4.5. RNA Extraction, cDNA Synthesis and qRT-PCR

Total RNA was extracted from tissue homogenates using the Spectrum^TM^ Plant Total RNA Kit (Sigma-Aldrich, ST. Louis, MO, USA), and treated with DNase I (New England Biolabs, Ipswich, MA, USA) as per the manufacturer’s protocol. RNA integrity was tested by agarose gel electrophoresis and spectrophotometry using a Thermo Scientific Nanodrop (Thermo Fisher Scientific, Waltham, MA, USA). DNase treated RNA (1 µg) was reverse transcribed using the Invitrogen Superscript IV RT (Invitrogen, CarlsbadCA, USA), according to the manufacturer’s instructions. Synthesized cDNAs were diluted 1:20 with RNase/DNase-free water. Fragments amplified using gene-specific primers designed to the 3’-untranslated regions (Table S3) were purified using HPLC following Burton [66] and sequenced at the Australian Genome Research Facility (Adelaide, South Australia). qRT-PCR was carried out following Burton [67]. Optimal acquisition temperatures are described in Table S3. The transcript levels of genes encoding actin (*ACTB*), cyclophilin (*CYP*), α-tubulin (*TUBA1A*) and elongation factor 1-α (*EFA*) were used as reference genes (Table S3).

### 4.6. Statistical Analysis

Gene expression data obtained from qRT-PCR, and hormone and anthocyanin data obtained from LC-MS/MS, were analyzed using a one-way ANOVA test coupled to a Waller-Duncan post hoc test using IBM SPSS statistical software. Post-harvest fruit parameter measurements were analyzed using a *t*-test on IBM SPSS. The level of significance was set at *p* < 0.05.

## Figures and Tables

**Table 1:** Effect of pre-ripening NAA treatment on fruit maturity parameters at harvest (13 January 2020, 42 dps).

Parameter	Control ^§^	NAA-Treated
Weight (g)	9.95 ± 0.075 **	11.2 ± 0.108 **
TSS (degrees Brix)	17.9 ± 0.406	18.8 ± 0.536
pH	3.93 ± 0.050	4.05 ± 0.026
Total acid (g/L) ^¶^	8.0 ± 0.233	7.3 ± 0.186
Cracking index	36.0 ± 4.365 **	8.0 ± 2.089 **
Firmness (g/mm)	357 ± 7.597 **	329 ± 4.393 **
L*	37.6 ± 0.745 **	30.6 ± 0.167 **
a*	15.7 ± 0.291 **	10.1 ± 0.521 **
b*	7.1 ± 0.115 **	4.1 ± 0.219 **
Chroma	17.3 ± 0.318 **	10.9 ± 0.555 **
Hue angle	24.3 ± 0.058 **	22.2 ± 0.367 **

**^§^** Values represent means ± standard error (*n* = 3) and ** denotes significant differences between control and NAA-treated samples (T-Test, *p* < 0.05). ^¶^ malic acid equivalents. dps = days post spray. TSS = total soluble solids. L* = lightness from black (0) to white (100), a* = from green (-) to red (+), b* = from blue (−) to yellow (+). Hue angle expresses the colour nuance [24] and values are defined as follows: red-purple: 0 degrees, yellow: 90 degrees, bluish-green: 180 degrees, and blue: 270 degrees [25]. The chroma, obtained as the square root of (a*^2^+b*^2^), is a measure of chromaticity (C*), which denotes the purity or saturation of the colour [24].

## Data Availability

Not applicable.

## Supplementary Materials

The following are available online at https://www.mdpi.com/article/10.3390/ijms221910760/s1, Figure S1: LC-MS/MS analysis of anthocyanins, Figure S2: LC-MS/MS analysis of NAA, Table S1: Anthocyanin LC-MS/MS transitions, fragmentor and collision energies, Table S2: Hormone LC-MS/MS run parameters, Table S3: Gene-specific qRT-PCR primers.
