# Supplementary material for: Auxin Treatment Enhances Anthocyanin Production in the Non-Climacteric Sweet Cherry (Prunus avium L.)
Source: Int J Mol Sci. 2021 Oct 5;22(19):10760. doi: 10.3390/ijms221910760 (PMC8509301; doi:10.3390/ijms221910760)
Supplement: Supplementary file 1 [file ijms-22-10760-s001.zip › ijms-1404992-supplementary.pdf]

Supplementary Material

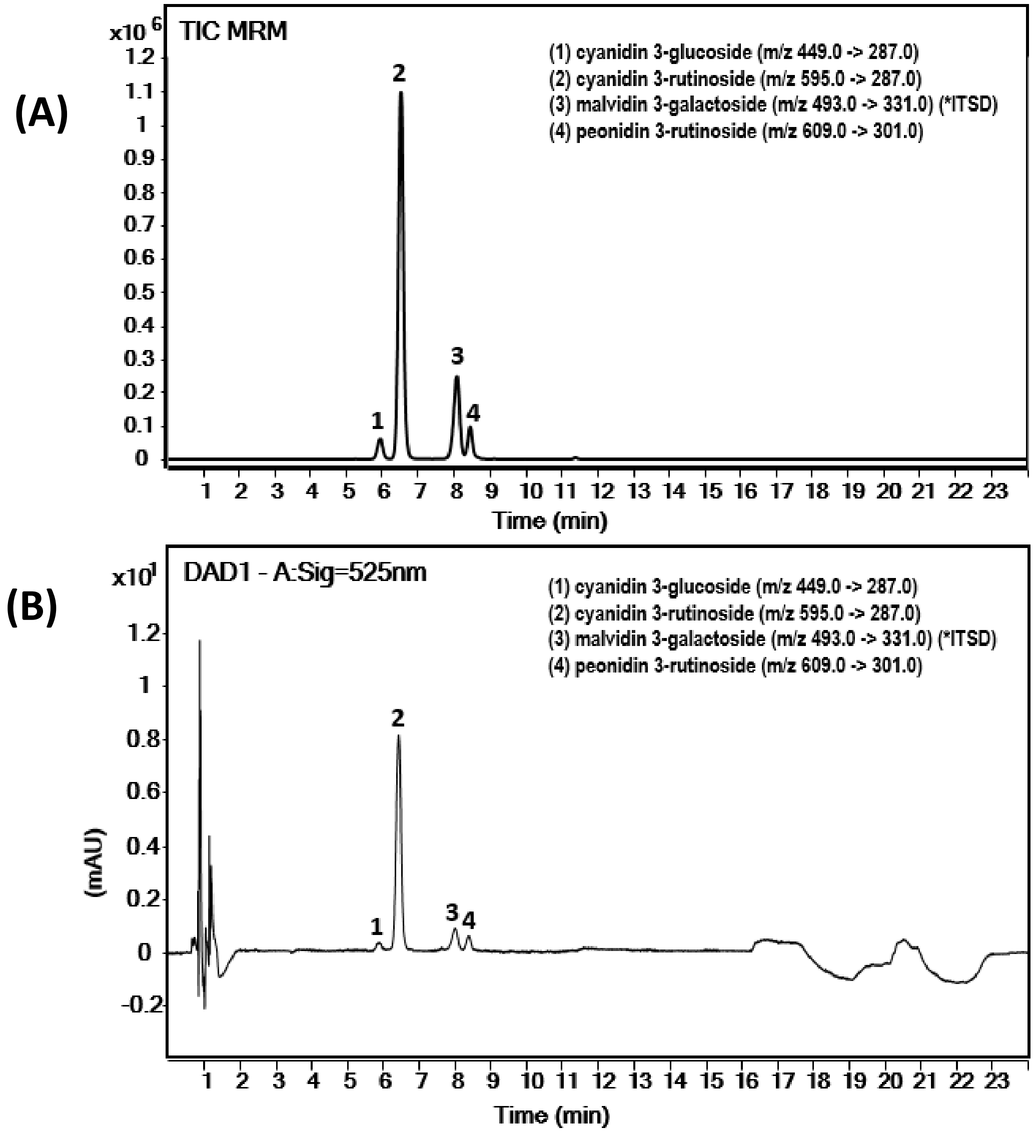

Figure S1. (**A**) LC-MS/MS MRM and (**B**) UV chromatograms of detectable anthocyanins found within the Sweet Georgia cherry variety and the anthocyanin used as an internal standard (malvidin 3-galactoside). TIC MRM: total ion chromatogram multiple reaction monitoring, DAD: diode array detector, mAU: milli-absorbance unit.

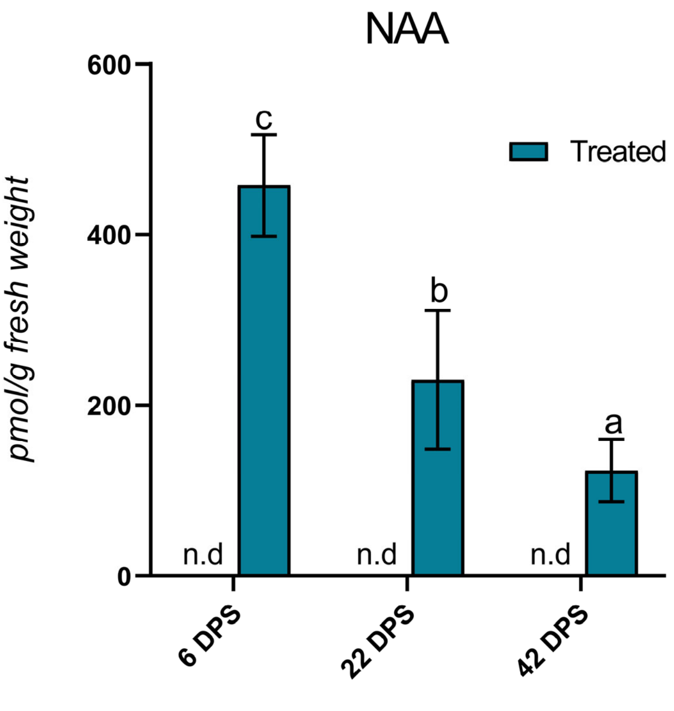

Figure S2. LC-MS/MS analysis of 1-naphthaleneacetic acid (NAA) concentration in sweet cherry (*Prunus avium* L.) fruit at three timepoints after the pre-ripening application of 100 mg/L NAA. All data represent means ± s. e. (*n* = 3), letters represent significant statistical differences of the mean values of NAA-treated samples from the mean values of control samples. n.d = not detected.

Table S1. Anthocyanin compounds, transitions, fragmentor and collision energy voltages used in LC-MS/MS analysis.

Compound	GasTemp (°C)	GasFlow (L/min)	Nebulizer (psi)	Sheath Gas Heater (°C)	Sheath Gas Flow (L/min)	Capillary (V)	Charging (V)	Precursor Ion MS [M-H]+	Product Ion MS/MS	Fragmentor (V)	Collision Energy (eV)
Peonidin 3-rutinoside	230	20	40	300	12	3000	500	609.0	301.0	166	22
Cyanidin 3-rutinoside	230	20	40	300	12	3000	500	595.5	287.0	166	22
Malvidin 3-galactoside	230	20	40	300	12	3000	500	493.0	331.0	166	22
Cyanidin 3-glucoside	230	20	40	300	12	3000	500	449.0	287.0	166	22

Table S2. LC-MS/MS run parameters, transitions, fragmentor and collision energy voltages of hormone compounds detectable in Sweet Georgia cherry.

Compound	Gas-Temp (°C)	GasFlow (L/min)	Nebulizer (psi)	Sheath Gas Heater (°C)	Sheath Gas Flow (L/min)	Capillary (V)	Charging (V)	Precursor Ion MS [M-H]-	Product Ion MS/MS	Fragmentor (v)	Collision Energy (eV)
ABA	300	8	35	350	11	2000	1200	263.1	219.1	86	12
ABA-GE	300	8	35	350	11	2000	1200	425.2	263.0	100	4
DPA	300	8	35	350	11	2000	1200	281.1	237.0	100	8
PA	300	8	35	350	11	2000	1200	279.1	205.1	106	12
ACC	300	8	55	350	11	3500	500	102.1	61.1	58	4
IAA	300	8	55	350	11	3500	500	176.1	144	68	4
IAA-Asp	300	8	55	350	11	3500	500	291.1	176.2	78	8

Table S3. Gene-specific qPCR primers, with PCR product sizes in base pairs and optimal acquisition temperatures for the genes analysed.

Gene	Forward Primer (5′-3′)	Reverse Primer (5′-3′)	Amplicon bp	Acquisition Temperature °C
CHS1	CAGGAGATGGACTGGATTGG	CGAAC-GAAACAGATGCATAAAA	148	77
CHS3	ATGTCGAGTGCTTGTGTGCTG	TACACCATGATGCATCTGGAAG	285	78
LDOX	CGAG-TATGCTAAGGAACTGAGG	TGAGGGCAAACTGGGTAGTAG	156	78
UFGT	TGTTGAGGATGGGGTTTTTAC	AG-TACAGCTCGGTTATTCTTATGC	243	80
DFR	CTTGGAGGACATGTTCGTAGG	AAAGAACCCAACAGACAC-TAATCC	189	81
PAL	ACAGCTATCTGCGAGGGAAA	CACAGTCTACCAGCAATGCAA	165	79
bHLH3	AGAGTGACGGGTTGTTGGAG	CCCTCAGCTCAGCTACGAAG	144	80
GST	TGTGAATGCTTGGTGGAAAG	TCTCAGTTCTTTCTTCCACATGA	124	78
EIN4	GGAGGAACATGTGTGGGAGA	TGTGCTCATTAGAGAGCCTGAA	153	79
ETR2	ATGTTTGCAGAGTGGCATCA	CCGCACAAGAGAGAAAAAGG	198	80
ACO	CTATGCTGGCGTCAAGTTCC	AAAGACTTTGGCCACACACA	171	78
ACS	CCAGGTTGGTTTAGGGTGTG	TGGGTAGGATCACAA-TAGGAAGA	154	80
PIN6	GTGAGGATGGTGTGGGAGTC	GACACCGTTTGTCCCCTTC	165	79
SAUR50	TTCTTGACTCGCCCTGAGTT	TCAGCCATTTTCTCCCAAAG	212	81
NCED1	TTCAACTACCCTCCAGAG-TTCC	GGGGGCGTATCAGATAGCTT	158	78
MYB10.1-1	ATCGTAATAAGAC-CTCAACCCCA	CTCGACCGTTTGAATATGGTCC	160	78
ACTB	AACTATGTTCCCCGGTATTGC	TGACATGACACGAAAATCCAA	282	80
CYP	CGGATCTCAGTTCTTCGTCTG	CCTAATCAGACCCCAGCCTAC	300	83
TUBA1A	TGCTCTGGAGAAGGATTACGA	AGCAGCAGCAAATAAAACCAA	247	78
EFA	CTGGTGCTAAGATCAC-CAAGG	GCCTAACAATGACGACCAAAA	185	78
